# Collaborative development of a scoping review protocol to map instruments assessing the parent–infant relationship: An International Initiative from COST Action TREASURE

**DOI:** 10.12688/openreseurope.21700.2

**Published:** 2026-01-16

**Authors:** Sónia Brandão, Anat Talmon, Ewa Gieysztor, Patrícia Souto, Andreia Soares Goncalves, Rosa Silva, Patrícia Gonçalves, Paula Prata, Özlem Şensoy, Esra Ardahan Akgül, Semra Pinar, Kristiina Uriko, Seda Ardahan Sevgili, Elif Bulut, Rajesh Shigdel, Demet Gülaldı, Otília Freitas, Aycin Ezgi Onel, Pelin Dikmen-Yildiz, Carmen Power, Alena Lochmannová, Dora d'Orsi, Özlem Koç, Tuğçe Sönmez, Tânia Brandão, Diana Azevedo, Lence Miloseva, Edanur Tar Bolacali, Bahar Aksoy, Mirlinda Markaj, Gilberta Sousa, Burcu Kömürcü Akik, Nicola Carone, Pinar Gencpinar, Ayça Demir Yıldırım, Hazal Özdemir Koyu, Wilson Abreu, Tuğba Yılmaz Esencan, Margarida Reis Santos, Mario Santos, Remziye Can, Anna-Lena Zietlow, Rafael Caparros-Gonzalez

**Affiliations:** 1 Nursing School of the University of Porto, Porto, 4200-072, Portugal; 2 RISE-Health, Nursing School of the University of Porto, Porto, 4200-072, Portugal; 3 Paul Baerwald School of Social Work and Social Welfare, Jerusalem, Jerusalem District, Israel; 4 Faculty of Physiotherapy, Wroclaw Medical University, Wrocław, Poland; 5 Polytechnic Institute of Viana do Castelo School of Health, Viana do Castelo, Viana do Castelo District, Portugal; 6 Health Sciences Research Unit, Nursing UICISA: E, Coimbra, Portugal; 7 Department of Pediatric Nursing, Istanbul University, School of Nursing, Istanbul, Turkey; 8 Izmir Katip Çelebi University, Faculty of Health Sciences, Department of Pediatric Nursing, Izmir, Turkey; 9 Department of Midwifery, Faculty of Health Sciences, Kahramanmaraş Sütçü Imam University, Kahramanmaraş, Turkey; 10 School of Psychology, Faculty of Medicine and Health, University of Leeds, Leeds, England, UK; 11 Department of Psychology and Behavioral Sciences, School of Natural Sciences and Health, Tallinn University, Tallinn, Estonia; 12 Ege University, Nursing Faculty, Department of Pediatric Nursing, İzmir, Turkey; 13 Tokat Gaziosmanpasa University, Faculty of Health Sciences, Department of Nursing, Child Health Nursing, Tokat, Turkey; 14 Department of Global Public Health and Primary Care, University of Bergen, Alrek helseklynge, blokk D, Årstadveien 175009, Bergen, Norway; 15 Department of Child Development, Uskudar University, Faculty of Health Science, Istanbul, Turkey; 16 RISE-HEALTH-UMa, RISE-HEALTH-UMa ; Escola Superior de Saúde da Universidade da Madeira, Funchal, Portugal; 17 Department of Psychology, Kirklareli University, Kirklareli, Turkey; 18 Independent Psychologist, Researcher, Educator, Consultant, Bristol, UK; 19 Department of Infectious Diseases and Travel Medicine, Faculty of Medicine in Pilsen, Charles University, Pilsen, Czech Republic; 20 William James Center for Research, Ispa–Instituto Universitário, Rua Jardim do Tabaco, 34, 1149-041 Lisbon, Portugal, Lisbon, Portugal; 21 Department of Midwifery, Tarsus University, Faculty of Health Sciences, Tarsus, Mersin Province, Turkey; 22 ICVS/3B's – PT Government Associate Laboratory, Braga/Guimarães, Portugal; 23 Department of Neuropsychiatry and Clinical Psychology, Faculty of Medical Sciences, Goce Delcev University Stip, Shtip, North Macedonia; 24 Department of Pediatric Nursing, Kirsehir Ahi Evran University, Faculty of Health Sciences, Kırşehir, Turkey; 25 Department of Pediatric Nursing, Akdeniz University, Kumluca Faculty of Health Sciences, Antalya, Turkey; 26 Department of Health Psychology, Comenius University in Bratislava, Faculty of Social and Economic Sciences, Bratislava, Bratislava Region, Slovakia; 27 Department of Psychology, Ankara University, Faculty of Languages and History-Geography, Ankara, Turkey; 28 Department of Systems Medicine, University of Rome Tor Vergata, Rome, Italy; 29 Department of Pediatric Neurology, Izmir Katip Celebi University Neuroscience Center, Izmir, İzmir, Turkey; 30 Department of Midwifery, Uskudar University, Faculty of Health Sciences, Istanbul, Turkey; 31 Gazi University, Nursing Faculty, Department of Pediatric Nursing,, Ankara, Turkey; 32 CARE - Research Center on Health and Social Sciences, Polytechnic Institute of Portalegre, Lisbon, Portugal; 33 Iscte - Instituto Universitário de Lisboa, Portalegre, Portugal; 34 Mustafa Kemal Atatürk Vocational and Technical High School, Eskişehir, Turkey; 35 Faculty of Psychology, Clinical Child and Adolescent Psychology, Dresden University of Technology, Dresden, Germany; 36 Instituto de Investigación Biosanitaria Ibs, Granada, Spain; 37 Departamento de Enfermería, Facultad de Ciencias de la Salud, Universidad de Granad, Granada, Spain

**Keywords:** parent–infant relationship; early relational health; scoping review; methodological framework; collaborative research; international collaboration; COST Action

## Abstract

Early relational health during the first 24 months of life is a key determinant of child development and wellbeing. During this postnatal period, the parent–infant relationship plays a central role in emotional regulation, bonding, and developmental trajectories. Although the broader early relational health framework encompasses the first 1,000 days of life, this scoping review focuses specifically on the postnatal phase, where parent–infant interactions are directly observable and measurable. However, existing assessment instruments vary widely in their conceptual focus, scope, and characteristics, and no comprehensive review has systematically mapped tools used to assess the parent–infant relationship during early infancy. In response to this gap, a transdisciplinary working group within the COST Action CA22114 – TREASURE collaboratively developed a scoping review protocol to systematically map instruments assessing the parent–infant relationship from birth to 24 months of age. This Brief Report describes the collaborative methodological process underpinning the protocol’s development. The process followed an iterative, consensus-driven approach involving multidisciplinary experts from multiple COST member countries. Through structured online meetings, the group clarified core constructs and established the age range using the Population–Concept–Context (PCC) framework. The JBI methodology for scoping reviews was adopted and aligned with PRISMA-ScR standards to ensure transparency and reproducibility. Progressive drafting, internal peer review, and iterative refinement led to the final protocol, which was registered on the Open Science Framework (DOI: 10.17605/OSF.IO/HRVX9).The resulting protocol provides a replicable methodological framework for mapping instruments that assess the parent–infant relationship in the first two years of life. This Brief Report presents a framework for collaborative protocol development in international research networks, promoting shared knowledge generation in early relational health research and offering potential applicability to other COST initiatives.

## Plain Language Summary

This article explains how an international team of researchers worked together to create a structured plan (protocol) for a scoping review, a type of research review that maps existing evidence. The aim of this review is to identify and describe the tools used to assess the relationship between parents and their babies during the first two years of life, a crucial period for emotional bonding, development, and long-term health. Experts from different countries within the COST network collaborated step by step to define key concepts, agree on suitable methods, and design a clear and transparent protocol. This protocol will guide a future scoping review that will identify and map all instruments used to evaluate the parent–infant relationship. The findings will help healthcare professionals, researchers, and policymakers better understand the range and characteristics of available instruments, supporting informed decisions about their use in promoting early relational health.

## Introduction

Early childhood development is widely recognised as a global public health priority, aligned with the Sustainable Development Goals and the Convention on the Rights of the Child. The first 1,000 days of life, from conception to a child’s second birthday, represent a critical period of rapid brain growth and heightened neuroplasticity ( Berg, 2016; Scher, 2024; Wardoyo *et al.*, 2024). Within this broad framework of early childhood development, increasing attention has been directed towards early relational health, the quality of early caregiving relationships that form the foundation for emotional, social, and cognitive development. During this time, interactions between infants and their primary caregivers shape neurocognitive, socioemotional, and self-regulatory development, with long-term implications for physical and mental health ( Black & Merseth, 2018; Scher, 2024). Nurturing and responsive caregiving environments promote adaptive developmental trajectories, whereas adverse or disrupted early relationships may contribute to stress dysregulation and increased vulnerability to later mental health difficulties ( Bhamani *et al.*, 2023).

Parental mental health during the perinatal period, particularly elevated stress, anxiety or depressive symptoms, can significantly influence how parents adapt to their new relational roles after birth ( Aktar *et al.*, 2019; Stein *et al.*, 2014). Stress-related physiological changes, such as hormonal, inflammatory, and neuroendocrine alterations, may not only affect pregnancy outcomes, including preterm birth or low birth weight, but also shape the conditions under which early parent–infant bonding begins ( Wardoyo *et al.*, 2024). These influences extend beyond individual parental wellbeing, affecting the quality of early interactions and the foundations of the parent–infant relationship in the postnatal period. The COST Action CA22114 – Maternal Perinatal Stress and Adverse Outcomes in the Offspring: Maximising Infants’ Development (TREASURE) was established to address these challenges by integrating a multidisciplinary European network to explore strategies that mitigate the effects of perinatal stress and promote early relational health. Within this framework, the parent–infant relationship is recognised as a key mediator linking perinatal stress with developmental outcomes ( Fredriksen *et al.*, 2019; Kim *et al.*, 2016). Sensitive and attuned interactions support secure attachment and healthy development, while disruptions in these relationships are linked to poorer outcomes ( Korom & Dozier, 2021; Wardoyo *et al.*, 2024). Accordingly, international health and research agendas increasingly recommend the systematic assessment of the parent–infant relationship within perinatal and early childhood care to support early identification of relational risk and timely intervention ( Bhamani *et al.*, 2023).

Several instruments have been developed to assess aspects of the parent–infant relationship, including bonding (e.g., Postpartum Bonding Questionnaire; Brockington *et al.*, 2006), attachment (e.g., Maternal Postnatal Attachment Scale; Condon & Corkindale, 1998), and interactional quality (e.g., Parent–Child Early Relational Assessment; Clark, 1999; Parent–Infant Interaction Observation Scale; Svanberg *et al.*, 2013). (e.g., Parent–Child Early Relational Assessment; Parent–Infant Interaction Observation Scale). However, existing measures differ widely in their conceptual focus, psychometric properties, and intended applications. Despite growing global interest, no scoping review to date has systematically mapped instruments that assess the parent–infant relationship specifically within the first 24 months of life.

The focus on the postnatal period up to 24 months is developmentally grounded, as this timeframe encompasses key transitions including the consolidation of attachment patterns, increasing infant autonomy, and qualitative changes in interactional modalities, all of which are central to early relational health. This Brief Report outlines the collaborative and transnational methodological process that guided the development of the scoping review protocol, offering a structured framework for evidence mapping within multidisciplinary international research networks. Its primary aim is to document the key steps and collaborative mechanisms that informed the protocol’s design.

## Methods

This Brief Report describes the collaborative process through which the scoping review protocol was developed within the COST Action CA22114. The methodological process followed distinct, iterative phases, detailed below.

### a) Collaborative framework design

The protocol was developed within Working Group 4.3 (WG4.3) of COST Action CA22114 – TREASURE, a multidisciplinary international research network addressing perinatal stress and early developmental outcomes. WG4.3 included researchers and clinicians from different countries participating in the Action, with expertise in maternal and child health nursing, midwifery, psychology, psychiatry, paediatrics, developmental sciences, and perinatal mental health. The development adopted a collaborative and transdisciplinary approach that integrated diverse professional perspectives, resulting in an internationally applicable and methodologically robust protocol.

### b) Consensus development process

The scoping review topic was identified through structured discussions held during early WG4.3 meetings, where members highlighted the need to map existing instruments assessing the parent–infant relationship. A structured consensus-building process was adopted, involving iterative online deliberations via a videoconferencing platform. During guided discussions, the group clarified conceptual boundaries (e.g., relationship, bonding, attachment, interaction, responsiveness) and discussed how conceptual overlap between these constructs would be addressed during data charting and synthesis. The group agreed to focus on instruments applicable to infants aged 0–24 months. The scope was defined using the Population–Concept–Context (PCC) framework recommended by the JBI ( Peters *et al.*, 2024): opulation = infants aged 0–24 months and their parents; Concept = instruments assessing the parent–infant relationship; Context = any setting, without geographical or clinical restrictions. Consensus was achieved through iterative negotiation and member validation at each stage.

### c) Methodological alignment and search strategy development

The JBI methodology for scoping reviews was adopted due to its suitability for mapping emerging evidence without restricting study design ( Peters *et al.*, 2024). The protocol was aligned with the Preferred Reporting Items for Systematic Reviews and Meta-Analyses extension for Scoping Reviews (PRISMA-ScR) to ensure transparency and reproducibility ( Tricco *et al.*, 2018).

The search strategy was collaboratively developed by the research team with methodological input from a health sciences librarian experienced in evidence synthesis. Guided by the PCC framework, keyword clusters were generated using both free-text terms and controlled vocabulary (e.g., MeSH in PubMed, Emtree in Embase). Iterative pilot searches in PubMed were conducted to refine term relevance, sensitivity, and specificity. Boolean logic (OR within clusters and AND between clusters) was applied to construct the final strategy. All keyword iterations and database-specific strings were documented to support transparency and replicability. The final search strategy was endorsed through group consensus before being incorporated into the registered protocol.

### d) Protocol development phases

Protocol drafting followed an iterative co-construction process using a shared online document platform, enabling simultaneous contributions and asynchronous revisions. The initial sections were developed using the JBI template and refined through structured internal feedback cycles. Special attention was given to clarifying operational definitions, eligibility criteria, and the overall methodological flow. Once full agreement was achieved, the protocol was registered on the Open Science Framework (OSF) (DOI: 10.17605/OSF.IO/HRVX9), ensuring transparency, traceability, and alignment with the open science principles that underpin COST-funded collaborations.

### e) Tools and platforms

A range of digital tools supported the collaborative development process. An online videoconferencing platform was used for synchronous consensus meetings, while a shared online document platform enabled iterative drafting and real-time commentary. Spreadsheet software facilitated initial screening tests and the organisation of database structures. Covidence was selected for the dual-reviewer screening workflow due to its structured interface for title, abstract, and full-text assessment. The OSF served as the public repository for protocol registration and documentation of methodological decisions, reinforcing transparency and adherence to open research principles.

### f) Pilot testing of screening procedures

A pilot validation exercise was conducted during the protocol development phase to ensure the clarity, feasibility, and consistency of the proposed screening tools and procedures. This pre-test did not constitute formal screening, but rather simulated the title and abstract screening process using a small, random subset of search results to verify the applicability of the PCC framework and to promote inter-reviewer alignment. Each reviewer independently applied the title and abstract screening tool (phase 1) to identify potential ambiguities and ensure shared understanding of inclusion and exclusion criteria.

Findings from this pre-test were discussed in a consensus meeting, leading to the refinement of the screening criteria and clarification of decision rules. Based on collective feedback, the title and abstract screening tool (phase 1) was revised, and a full-text screening tool (phase 2) was developed for use in the subsequent implementation of the scoping review.

This structured and iterative validation process strengthened the internal consistency and usability of the protocol and ensured methodological readiness for the forthcoming scoping review. Collectively, these steps illustrate a rigorous and transparent process of protocol development that underpins the collaborative framework described next.

## Results

### Results – Output of the collaborative process

The collaborative work of WG4.3 culminated in the articulation of a structured collaborative workflow that captures the stages, roles, and decision-making processes involved in the development of the scoping review protocol. This framework integrates the agreed conceptual parameters (PCC), methodological alignment with JBI and PRISMA-ScR guidance, and the collaborative mechanisms that guided protocol construction. Rather than presenting empirical results, this output represents the methodological product of the collaborative process, reflecting a transparent and consensus-based workflow that can be adapted for other multidisciplinary and international research initiatives.

**Figure 1.**
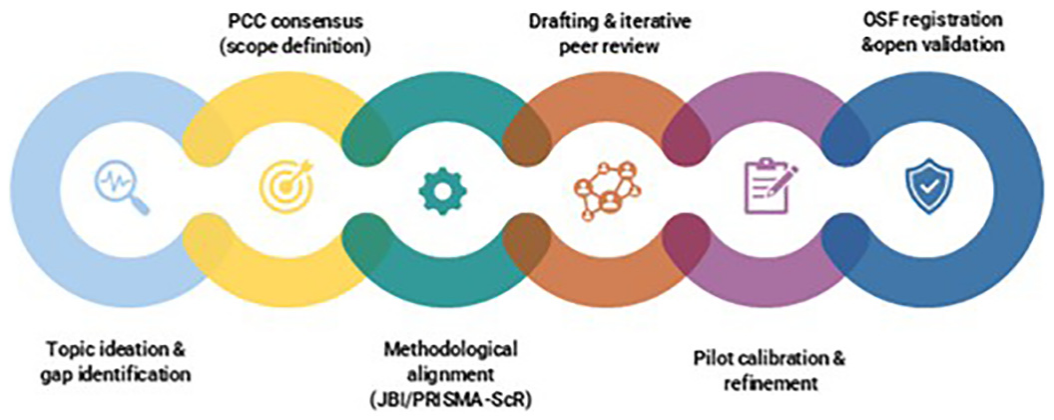
Stages of the collaborative workflow used to develop the scoping review protocol. The figure illustrates the sequential stages of the collaborative process, including initial ideation, definition of the Population–Concept–Context (PCC) framework, theoretical and methodological alignment with JBI and PRISMA-ScR guidance, protocol drafting, iterative collaborative review, and registration on the Open Science Framework (OSF). Standard scoping review development phases are integrated with network-specific collaborative elements characteristic of COST Action–based research.

The final collaborative workflow is summarised in Figure 1.

## Conclusions/Discussion

This scoping review protocol was conceived in response to the growing recognition of the parent–infant relationship as a foundational determinant of health during the first 1,000 days of life. In this context, mapping the available assessment instruments focused on the postnatal period is essential to guide research, clinical practice, and early intervention strategies, since existing measures vary in their scope, conceptual foundations, and methodological characteristics. By formalising a rigorously developed protocol, this initiative addresses a critical gap and establishes a structured pathway for synthesising and describing evidence on instruments assessing the parent–infant relationship in infants aged 0–24 months.

A key strength of this work lies in its explicitly collaborative, transdisciplinary, and international nature, embedded within the COST Action CA22114 – TREASURE network. The contribution of experts enabled conceptual convergence across diverse perspectives on bonding, attachment, interaction, and responsiveness. The structured consensus process fostered shared ownership of methodological decisions, enhancing the protocol’s international applicability and adaptability.

Methodological rigour was ensured through alignment with established standards (JBI framework; PRISMA-ScR), open registration on the OSF, and a pilot validation phase conducted during the protocol development, which improved inter-reviewer consistency and clarified screening criteria.

Beyond establishing a methodological foundation, this protocol sets the stage for identifying conceptual, psychometric, and cultural gaps in existing instruments. Mapping tools according to their constructs, domains, reliability, validity, usability, and contextual adaptation will inform both research and practice, while also guiding future tool development, particularly in relation to paternal involvement, cultural sensitivity, and dynamic interaction-based assessments. While embodied and interaction-based dimensions of early relationships are increasingly recognised as important, particularly in vulnerable populations such as preterm infants ( La Rosa *et al.*, 2024), these populations fall outside the scope of the present protocol. Future reviews may build on this work by specifically addressing embodied relational processes in high-risk or neonatal contexts. Ultimately, this Brief Report presents not only the outcome of a protocol development process but also a replicable framework for collaborative methodological construction within international research networks. The protocol, titled “Instruments for the assessment of parent–infant relationships: A scoping review protocol” and registered on OSF, provides the foundation for a forthcoming scoping review that will generate insights into the availability, conceptual diversity, and psychometric adequacy of relational assessment tools in early infancy.

In conclusion, this work demonstrates how structured, consensus-based collaboration within COST networks can produce methodologically sound and widely applicable research frameworks. By clarifying the field of parent–infant relational assessment, it contributes to advancing research, clinical decision-making, and policy development in early relational health, offering a scalable model for future interdisciplinary evidence synthesis initiatives across diverse international contexts.

## Ethics and consent

Ethical approval and consent were not required for this study, as it reports the collaborative development process for a scoping review protocol and does not involve human participants or personal data.

## Data Availability

No underlying or extended data are associated with this manuscript, as it documents the collaborative process of protocol development rather than reporting research findings. The scoping review protocol described in this Brief Report is openly available on the Open Science Framework (OSF): https://doi.org/10.17605/OSF.IO/HRVX9 ( Brandão *et al.*, 2025). Data are available under the terms of the Creative Commons Attribution 4.0 International license (CC-BY 4.0).

## References

[ref-1] Aktar E Qu J Lawrence PJ : Fetal and infant outcomes in the offspring of parents with perinatal mental disorders: earliest influences. *Front Psychiatry.* 2019;10:391. 10.3389/fpsyt.2019.00391 31316398 PMC6610252

[ref-2] Berg A : The importance of the first 1,000 days of life. *J Child Adolesc Ment Health.* 2016;28(2):iii–vi. 10.2989/17280583.2016.1223803 27562006

[ref-3] Bhamani S Syed A Sheikh L : Investing in mental health during the first 1,000 days of life: now and future. *J Pak Med Assoc.* 2023;73(2):374–376. 10.47391/JPMA.6044 36800729

[ref-4] Black M Merseth KA : First 1000 days and beyond: strategies to achieve the Sustainable Development Goals.In: S. Verma & A. Petersen (Eds.), *Developmental science and sustainable development goals for children and youth.*(Social Indicators Research Series), Springer,2018;74:97–112. 10.1007/978-3-319-96592-5_5

[ref-17] Brandão S Talmon A Gieysztor E : Instruments for the assessment of parent-infant relationships: a scoping review protocol. 2025. 10.17605/OSF.IO/HRVX9

[ref-5] Brockington IF Fraser C Wilson D : The postpartum bonding questionnaire: a validation. *Arch Womens Ment Health.* 2006;9(5):233–242. 10.1007/s00737-006-0132-1 16673041

[ref-6] Clark R : The Parent–Child early relational assessment: a factorial validity study. *Educ Psychol Meas.* 1999;59(5):821–846. 10.1177/00131649921970161

[ref-7] Condon J Corkindale C : The assessment of parent-to-infant attachment: development of a self-report questionnaire instrument. *J Reprod Infant Psychol.* 1998;16(1):57–76. 10.1080/02646839808404558

[ref-8] Fredriksen E von Soest T Smith L : Parenting stress plays a mediating role in the prediction of early child development from both parents’ perinatal depressive symptoms. *J Abnorm Child Psychol.* 2019;47(1):149–164. 10.1007/s10802-018-0428-4 29623542

[ref-9] Kim M Kang SK Yee B : Paternal involvement and early infant neurodevelopment: the mediation role of maternal parenting stress. *BMC Pediatr.* 2016;16(1): 212. 10.1186/s12887-016-0747-y 27955632 PMC5153858

[ref-10] Korom M Dozier M : The importance of responsive parenting for vulnerable infants.In: J. B. Benson (Ed.), *Adv Child Dev Behav.*Elsevier,2021;61:43–71. 10.1016/bs.acdb.2021.03.001 34266571

[ref-18] La Rosa VL Geraci A Iacono A : Affective touch in preterm infant development: Neurobiological mechanisms and implications for child–caregiver attachment and neonatal care. *Children (Basel).* 2024;11(11): 1407. 10.3390/children11111407 39594981 PMC11592606

[ref-11] Peters MDJ Godfrey C McInerney P : Scoping reviews.In: E. Aromataris & C. Lockwood (Eds.), *JBI manual for evidence synthesis.*(Chap. 10). JBI,2024. 10.46658/JBIMES-24-09

[ref-12] Scher MS : The first 1000 days influence life-course brain health: interdisciplinary fetal/neonatal neurology training. *Pediatr Res.* 2024;96(4):838–840. 10.1038/s41390-022-01936-w 35173298

[ref-13] Stein A Pearson RM Goodman SH : Effects of perinatal mental disorders on the fetus and child. *Lancet.* 2014;384(9956):1800–1819. 10.1016/S0140-6736(14)61277-0 25455250

[ref-14] Svanberg PO Barlow J Tigbe W : The Parent–Infant interaction observation scale: reliability and validity of a screening tool. *J Reprod Infant Psychol.* 2013;31(1):5–14. 10.1080/02646838.2012.751586

[ref-15] Tricco AC Lillie E Zarin W : PRISMA extension for Scoping Reviews (PRISMA-ScR): checklist and explanation. *Ann Intern Med.* 2018;169(7):467–473. 10.7326/M18-0850 30178033

[ref-16] Wardoyo H Moeloek ND Basrowi RW : Mental health awareness and promotion during the first 1000 days of life: an expert consensus. *Healthcare (Basel).* 2024;12(1):44. 10.3390/healthcare12010044 38200950 PMC10778627

