# Supplementary material for: Collaborative development of a scoping review protocol to map instruments assessing the parent–infant relationship: An International Initiative from COST Action TREASURE
Source: Open Res Eur. 2026 Jan 16;5:394. Originally published 2025 Dec 19. [Version 2] doi: 10.12688/openreseurope.21700.2 (PMC12816950; doi:10.12688/openreseurope.21700.2)
Supplement: Peer review history

# Reviewer report: Reviewer response for version 2

**DOI:** 10.21956/openreseurope.24584.r68177

**Published:** 2026-01-19

**Authors:** Valentina Lucia La Rosa

**Affiliations:** 1 Department of Educational Sciences, University of Catania, Catania, Sicily, Italy

I commend the authors for their careful revisions and for enhancing the overall readability and scientific quality of the manuscript. I have no further major or minor concerns, and I recommend the paper for Indexing.

Is the study design appropriate and does the work have academic merit?

Yes

Is the work clearly and accurately presented and does it cite the current literature?

Yes

If applicable, is the statistical analysis and its interpretation appropriate?

Not applicable

Are all the source data underlying the results available to ensure full reproducibility?

No source data required

Are the conclusions drawn adequately supported by the results?

Yes

Are sufficient details of methods and analysis provided to allow replication by others?

Yes

Reviewer Expertise:

Developmental Psychology

I confirm that I have read this submission and believe that I have an appropriate level of expertise to confirm that it is of an acceptable scientific standard.

# Reviewer report: Reviewer response for version 1

**DOI:** 10.21956/openreseurope.23474.r67323

**Published:** 2026-01-10

**Authors:** Dean McDonnell

**Affiliations:** 1 South East Technological University, Waterford, County Waterford, Ireland

The report authored by Brandão et al outlines the collaborative, transdisciplinary development of a protocol designed to map instruments assessing the parent–infant relationship from birth to 24 months. Developed within the merits of the COST Action CA22114 (TREASURE), the paper presents the procedural process rather than results, which greatly contributes to and aligns with consensus-building, methodological alignment, and open science principles, including OSF registration.

 The manuscript is clearly written, well-structured, and aligns with the aims of *Open Research Europe* in terms of transparency, collaboration, and methodological rigour. Its primary contribution lies in offering a framework that is replicable, especially important when factoring the context of international collaboration, rather than advancing new empirical findings. This is appropriate for the article type, but also creates several areas where clarity, justification, and critical reflection could be strengthened.

 The main strengths I would see here are the documents clear rationale and relevance to international policy, the methodological clarity and rigour, the underlying ethos of open science and collaboration, and its overarching scope within the discipline.

 In terms of improvements, it may be useful to further evidence why there is need for a more explicit process to be documented (I fully understand why, but other academics/researchers may not), taking into account cultural developments, or conflicts within paradigms, operational definitions or terminology. It may also be useful to indicate how areas of attachment, parental bonding, and responsiveness were framed and discussed within the initial stages of the process.

 Overall, I would be very happy to approve this piece with some minor clarifications to the above.

Is the study design appropriate and does the work have academic merit?

Yes

Is the work clearly and accurately presented and does it cite the current literature?

Yes

If applicable, is the statistical analysis and its interpretation appropriate?

Not applicable

Are all the source data underlying the results available to ensure full reproducibility?

No source data required

Are the conclusions drawn adequately supported by the results?

Yes

Are sufficient details of methods and analysis provided to allow replication by others?

Yes

Reviewer Expertise:

Developmental psychology, interpersonal relationships, technology interaction, research methodology.

I confirm that I have read this submission and believe that I have an appropriate level of expertise to confirm that it is of an acceptable scientific standard.

# Response

**Published:** 2026-01-10

**Authors:** Sónia Brandão

**Affiliations:** Escola Superior de Enfermagem da Universidade do Porto, Porto, Porto District, Portugal

We sincerely thank the reviewer for his careful reading of the manuscript and for his positive and constructive assessment. We particularly appreciate the recognition of the manuscript’s alignment with the principles of transparency, collaboration, methodological rigour, and open science promoted by Open Research Europe, as well as the acknowledgment of its contribution as a replicable methodological framework within an international research context. We also thank the reviewer for the thoughtful suggestions aimed at strengthening the clarity, justification, and critical reflection underpinning the documented collaborative process. In response, we have made the following revisions to the manuscript: Rationale for documenting the collaborative process To further clarify why the explicit documentation of the collaborative protocol development process is necessary, we have strengthened the Introduction and Discussion to more clearly articulate the added value of this approach. In particular, we now emphasise how divergent conceptual traditions, operational definitions, and disciplinary perspectives within early relational health research necessitate structured, transparent, and consensus-based methodological processes, especially in large international and multidisciplinary networks. This clarification aims to make the relevance of documenting the process more explicit for readers who may be less familiar with the challenges inherent in such collaborations. Framing of attachment, bonding, interaction, and responsiveness in the early stages In response to the reviewer’s suggestion, we have expanded the description of the initial consensus-building stages in the Methods section. We now explicitly describe how key relational constructs, including attachment, parental bonding, interaction, and responsiveness, were discussed, defined, and conceptually framed during the early phases of protocol development. This addition clarifies how areas of conceptual convergence and divergence were addressed and how shared definitions informed subsequent methodological decisions. These revisions were made with the aim of enhancing transparency, conceptual clarity, and critical reflection, while remaining consistent with the article type and scope of a Brief Report focused on methodological development rather than empirical outcomes. Once again, we thank the reviewer for his supportive evaluation and insightful recommendations, which have helped strengthen the manuscript’s clarity, justification, and relevance for an international academic audience.

# Reviewer report: Reviewer response for version 1

**DOI:** 10.21956/openreseurope.23474.r66853

**Published:** 2026-01-06

**Authors:** Valentina Lucia La Rosa

**Affiliations:** 1 Department of Educational Sciences, University of Catania, Catania, Sicily, Italy

This manuscript addresses an important and underexplored methodological dimension of early relational health research by documenting the collaborative construction of a scoping review protocol within a large international network. The paper is generally clear, well structured, and coherent with its stated aims. The following comments are intended to strengthen the manuscript further and improve its clarity and contribution.

 Major Revisions

  

1. Although the manuscript acknowledges the various constructs related to the parent-infant relationship, including bonding, attachment, interaction, and responsiveness, the conceptual differences between these constructs are not clearly defined. Since the scoping review aims to map instruments across these domains, including a concise integrative paragraph that clarifies how these constructs are theoretically distinguished and how overlaps will be handled during data extraction and synthesis would be beneficial. This clarification would strengthen the conceptual coherence of the protocol and reduce ambiguity for readers who are less familiar with this literature.
2. Focusing on the first 24 months is a reasonable choice that is consistent with the early relational health framework. However, the rationale for this cutoff should be more explicitly stated, particularly in relation to key developmental transitions, such as the emergence of attachment patterns, increasing infant autonomy, and changes in interactional modalities. Providing a brief developmental justification would reinforce the protocol's underlying methodological decisions.
3. Although the authors claim that no scoping review has systematically mapped instruments that assess the parent–infant relationship within the first 24 months, this assertion would be strengthened by a clearer positioning in relation to adjacent reviews or frameworks. Clarifying how this protocol differs from or builds upon previous reviews focused on bonding, attachment, or interactional measures would help readers better understand its novelty and scope.
4. The manuscript emphasizes the importance of mapping assessment tools for research and clinical practice. However, the implications for early intervention and preventive strategies could be articulated more clearly. Linking the availability and characteristics of instruments explicitly to their potential use in identifying relational risk or informing intervention pathways would strengthen the work's applied relevance.
5. In this context, it may be helpful to encourage more explicit consideration of instruments that capture the embodied and interactive dimensions of early relationships. This is particularly important for vulnerable populations, such as preterm infants, for whom early tactile and relational processes play a critical role in bonding and regulation. *(La Rosa et al., 2024)*

Minor Revisions

1. Some sections of the Introduction cover similar concepts related to early relational health and perinatal mental health. Streamlining these sections slightly would improve readability without compromising content.
2. Consistently use terms such as "parent–infant relationship", "early relational health", and related constructs throughout the manuscript, especially when transitioning between the conceptual and methodological sections.
3. Figure 1 is conceptually useful, but its caption could be slightly expanded to guide the reader more explicitly through how the stages relate to standard scoping review development phases versus network-specific collaborative elements.

Is the study design appropriate and does the work have academic merit?

Yes

Is the work clearly and accurately presented and does it cite the current literature?

Yes

If applicable, is the statistical analysis and its interpretation appropriate?

Not applicable

Are all the source data underlying the results available to ensure full reproducibility?

No source data required

Are the conclusions drawn adequately supported by the results?

Yes

Are sufficient details of methods and analysis provided to allow replication by others?

Yes

Reviewer Expertise:

Developmental Psychology

I confirm that I have read this submission and believe that I have an appropriate level of expertise to confirm that it is of an acceptable scientific standard, however I have significant reservations, as outlined above.

## References

[1] : Affective Touch in Preterm Infant Development: Neurobiological Mechanisms and Implications for Child–Caregiver Attachment and Neonatal Care. *Children* .2024;11(11) : 10.3390/children11111407 10.3390/children11111407 PMC1159260639594981

# Response

**Published:** 2026-01-10

**Authors:** Sónia Brandão

**Affiliations:** Escola Superior de Enfermagem da Universidade do Porto, Porto, Porto District, Portugal

Response to Reviewer: Valentina Lucia La Rosa We sincerely thank the reviewer for her thoughtful, detailed, and constructive evaluation of our manuscript. We greatly appreciate the recognition of the manuscript’s relevance and methodological contribution, as well as the insightful suggestions provided to further strengthen its conceptual clarity and applied relevance. Below, we outline how each of the reviewer’s comments has been addressed.

 Major comments 1. Conceptual distinction between bonding, attachment, interaction, and responsiveness We agree that greater conceptual clarity regarding these related constructs is essential. In response, we have revised the Introduction to include a concise integrative paragraph that distinguishes bonding, attachment, interaction, and responsiveness based on their theoretical foundations and typical modes of assessment. We also clarify how overlaps between constructs will be handled during data extraction and synthesis, ensuring transparent mapping while preserving conceptual distinctions. This addition aims to enhance coherence and reduce ambiguity for readers less familiar with this literature.

 2. Rationale for the 24-month cutoff We thank the reviewer for highlighting the need for a clearer developmental justification. The Introduction has been strengthened to explicitly explain the focus on the first 24 months in relation to key developmental transitions, including the consolidation of attachment patterns, increasing infant autonomy, and qualitative changes in interactional modalities. We also clarify that, while early relational health is often framed within the broader “first 1,000 days” perspective, this scoping review intentionally focuses on the postnatal period, where parent–infant interactions are directly observable and assessable.

 3. Positioning in relation to adjacent reviews To address this comment, we have refined the Introduction to more clearly position the protocol in relation to existing reviews focused on specific dimensions such as bonding, attachment, or interaction. We explicitly state how the present protocol differs by offering a comprehensive mapping of instruments across constructs within a defined developmental window (birth to 24 months), thereby clarifying its novelty and broader scope.

 4. Implications for early intervention and preventive strategies We agree that the applied relevance of this work can be articulated more clearly. The Discussion has been revised to explicitly link the mapping of assessment instruments to their potential role in identifying relational risk, informing early intervention pathways, and supporting preventive strategies in clinical and community settings.

 5. Embodied and interactive dimensions; vulnerable populations We appreciate this important suggestion. The revised Discussion now explicitly acknowledges the relevance of instruments that capture embodied and interactive dimensions of early relationships. While the scoping review protocol focuses on healthy infants and excludes preterm populations, we note the importance of such dimensions for understanding relational processes more broadly and identify this as a relevant consideration for future research and tool development, citing La Rosa et al. (2024) accordingly.   Minor comments 1. Streamlining overlapping sections We have streamlined sections of the Introduction to reduce redundancy between discussions of early relational health and perinatal mental health, improving readability while maintaining conceptual continuity. 2. Terminological consistency We have reviewed the manuscript to ensure consistent use of key terms, including “parent–infant relationship” and “early relational health,” particularly when transitioning between conceptual and methodological sections. 3. Figure 1 caption The caption of Figure 1 has been expanded to more clearly guide readers through the stages of the collaborative workflow and to distinguish standard scoping review development phases from network-specific collaborative elements.   Once again, we thank the reviewer for her insightful feedback, which has substantially strengthened the conceptual clarity, methodological transparency, and applied relevance of the manuscript.
